# Comparative Antifungal Activity of Medicinal Plant Extracts and Essential Oils Against Clinical Isolates of *Candida albicans* from Denture Stomatitis Patients

**DOI:** 10.3390/plants15091392

**Published:** 2026-05-01

**Authors:** Nazanin Fathi, Joo-Hyun Hong, Farzaneh Lotfipour, Samin Ghaffari, Reza Abbasi, Parina Asgharian, Rana Attaran, Hamed Hamishehkar, Maryam Kouhsoltani, Ki Hyun Kim

**Affiliations:** 1 Research Center for Immunodeficiencies, Pediatrics Center of Excellence, Children’s Medical Center, Tehran University of Medical Sciences, Tehran 1416753955, Iran; nazaninfathi26@gmail.com; 2 School of Pharmacy, Sungkyunkwan University, Suwon 16419, Republic of Korea; ehong@skku.edu; 3 Food and Drug Safety Research Center, and Department of Pharmaceutical and Food Control, Faculty of Pharmacy, Tabriz University of Medical Sciences, Tabriz 14766-51664, Iran; farzaneh.lotfipour@gmail.com; 4 Department of Orthodontics, Faculty of Dentistry, Shahid Beheshti University of Medical Sciences, Tehran 1985717413, Iran; saminghaffari@gmail.com; 5 Department of Oral and Maxillofacial Pathology, Faculty of Dentistry, Tabriz University of Medical Sciences, Tabriz 14766-51664, Iran; reza.abbasy20@gmail.com (R.A.); rana.ataran@gmail.com (R.A.); 6 Department of Pharmacognosy, Faculty of Pharmacy, Tabriz University of Medical Sciences, Tabriz 14766-51664, Iran; parina.asgharian@gmail.com; 7 Drug Applied Research Center, Tabriz University of Medical Sciences, Tabriz 14766-51664, Iran; hamishehkar.hamed@gmail.com; 8 New Material and Green Chemistry Research Center, Khazar University, 41 Mehseti Street, Baku AZ1096, Azerbaijan

**Keywords:** denture stomatitis, *Candida albicans*, *Zingiber officinale*, minimum inhibitory concentrations, antifungal activity

## Abstract

In this study, we investigated the antifungal potential of methanolic extracts and essential oils obtained from five medicinal plants (*Salvadora persica*, *Mentha spicata*, *Achillea millefolium*, *Matricaria chamomilla*, and *Zingiber officinale*) against 25 clinical isolates of *Candida albicans* collected from patients with denture stomatitis. Antifungal susceptibility was assessed using broth microdilution as the primary method, with agar diffusion assays performed to provide complementary visual confirmation. Nystatin was included as a reference control. Across the tested samples, essential oils consistently showed stronger antifungal effects than the corresponding methanolic extracts. Notably, *Z. officinale* essential oil exhibited the highest level of activity, inhibiting 15 out of 25 isolates and, in several cases, demonstrating efficacy comparable to or exceeding that of nystatin. Chemical profiling by GC–MS indicated that the ginger essential oil was dominated by sesquiterpene and monoterpene hydrocarbons, with zingiberene (21.49%) being the major constituent, followed by β-sesquiphellandrene, α-curcumene, sabinene, and α-citral. This terpene-rich composition may contribute to the observed antifungal activity, potentially through the disruption of fungal cell membrane integrity. Taken together, these results suggest that *Z. officinale* essential oil represents a promising natural antifungal candidate for the management of denture-associated *C. albicans* infections. Further studies, including biofilm-based assays and in vivo evaluations, will be necessary to confirm its clinical applicability. To the best of our knowledge, this study is among the first to comparatively assess these five medicinal plants against clinical *C. albicans* isolates derived specifically from denture stomatitis patients.

## 1. Introduction

Denture stomatitis (DS) is a common condition affecting up to 65% of denture wearers and is primarily associated with colonization by *Candida albicans*, often promoted by mechanical trauma or inadequate denture hygiene [1]. Clinically, DS is classified into three forms, namely localized inflammation (Type I), diffuse erythema (Type II), and inflammatory papillary hyperplasia (Type III), reflecting the increasing severity of mucosal involvement. The development of DS is multifactorial. In addition to mechanical irritation caused by poorly fitting dentures, reduced salivary flow and compromised local immune responses contribute to disease progression. A key feature of DS is the formation of *Candida* biofilms on denture acrylic surfaces. These biofilms provide a protective environment that enhances fungal survival and significantly reduces susceptibility to antifungal agents. This reduced sensitivity is largely attributed to the presence of an extracellular matrix, metabolic adaptation, and the activation of efflux pump systems. Compared with planktonic cells, *Candida* cells within biofilms are considerably more resistant to antifungal treatment, making infections difficult to eradicate. Given that *C. albicans* is also an important opportunistic pathogen in both superficial and systemic infections, its ability to form biofilms—particularly on porous denture materials—represents a major clinical challenge [1,2]. Therefore, strategies aimed at preventing or disrupting biofilm formation are of considerable importance, highlighting the need for the development of new antifungal agents.

Nystatin remains one of the most commonly prescribed antifungal agents for *C. albicans* infections, primarily exerting its effect through binding to ergosterol and disrupting fungal cell membranes. Despite its widespread use, several limitations restrict its clinical effectiveness. These include poor aqueous solubility, chemical instability, and the emergence of drug resistance among *Candida* strains [3,4]. In addition, nystatin therapy is often associated with practical drawbacks such as an unpleasant taste, the need for frequent administration, and limited efficacy against biofilm-associated infections. Recurrence of infection following treatment discontinuation is also frequently reported, further reducing its long-term therapeutic value and patient compliance. In light of these challenges, there has been growing interest in alternative therapeutic approaches, particularly the use of plant-derived compounds and herbal medicines as potential antifungal agents. Several herbs, including *Salvadora persica*, *Mentha spicata*, *Achillea millefolium*, *Matricaria chamomilla* (chamomile), and *Zingiber officinale* (ginger), have shown antimicrobial, anti-inflammatory, and analgesic properties. *S*. *persica* extract, commonly used as Miswak, has demonstrated antiplaque activity comparable to chlorhexidine gluconate [5]. Similarly, *M*. *spicata* and *A*. *millefolium* have shown both antibacterial and antifungal effects, with the essential oil of *A. millefolium* reported to be active against a range of pathogens, including *C. albicans* [6,7]. *M*. *chamomilla* is also well known for its antimicrobial and antioxidant properties, and its extracts have exhibited antifungal activity in vitro [8,9,10]. In addition, *Z*. *officinale* has attracted attention due to its broad-spectrum antimicrobial potential and emerging pharmaceutical applications. The biological activity of essential oils is often attributed to their hydrophobic constituents, which can interact with lipid bilayers and compromise membrane integrity, ultimately leading to cellular dysfunction. Moreover, the complex composition of these oils may contribute to reduced susceptibility to resistance development compared with conventional single-target agents. Despite these promising findings, direct comparative evaluations of these medicinal plants (particularly, their essential oils) against clinical *C. albicans* isolates obtained from denture stomatitis patients remain limited. Considering the increasing prevalence of antifungal resistance and the protective nature of biofilm-associated infections, systematic investigation of plant-derived antifungal agents under standardized conditions is therefore warranted.

As part of our ongoing efforts to discover bioactive natural products from natural resources [11,12,13,14,15], this study aims to evaluate the antifungal efficacy of *S. persica*, *M. spicata*, *A. millefolium*, *M. chamomilla*, and *Z. officinale* against clinical isolates of *C. albicans*, comparing their effects with that of nystatin. Importantly, antifungal susceptibility patterns of clinical isolates obtained from denture stomatitis patients may differ significantly from laboratory reference strains. Therefore, evaluating plant-derived antifungal agents against clinically relevant isolates provides more translationally meaningful data.

## 2. Results

### 2.1. Antifungal Susceptibility of Clinical Isolates of C. albicans

The antifungal susceptibility of 25 clinical isolates of *Candida albicans* obtained from denture stomatitis patients was evaluated against five plant extracts and their corresponding essential oils. The minimum inhibitory concentration (MIC) values for each isolate are summarized in Table 1. Among the tested isolates, sample no. 11 demonstrated sensitivity to all evaluated extracts and essential oils. In contrast, the remaining isolates exhibited selective sensitivity to certain preparations or showed no detectable response.

### 2.2. Comparative Antifungal Activity of Plant Extracts and Essential Oils

The number of susceptible isolates varied depending on the tested preparation. *Salvadora persica* extract showed limited activity, inhibiting the fungal growth of only two from the 25 isolates. *Achillea millefolium* essential oil and extract inhibited the growth of three and four isolates, respectively, while *Matricaria chamomilla* extract was active against four isolates. *Mentha spicata* essential oil showed antifungal activity against six isolates. In contrast, *Zingiber officinale* preparations demonstrated markedly stronger effects. Both the extract and essential oil inhibited 15 out of 25 isolates, representing the highest response rate among all tested samples. Overall, essential oils consistently exhibited greater antifungal activity than their corresponding methanolic extracts.

### 2.3. MIC Distribution and Comparison with Nystatin

Analysis of the MIC data showed that ginger essential oil had the lowest mean MIC among the tested plant preparations. In seven *C. albicans* isolates, its MIC values were comparable to or lower than those of nystatin used as the positive control. These results indicate that ginger essential oil exhibited the strongest anti-*C. albicans* activity among the evaluated natural products.

### 2.4. Confirmation by Agar Disc Diffusion Assay

**Figure 1:**
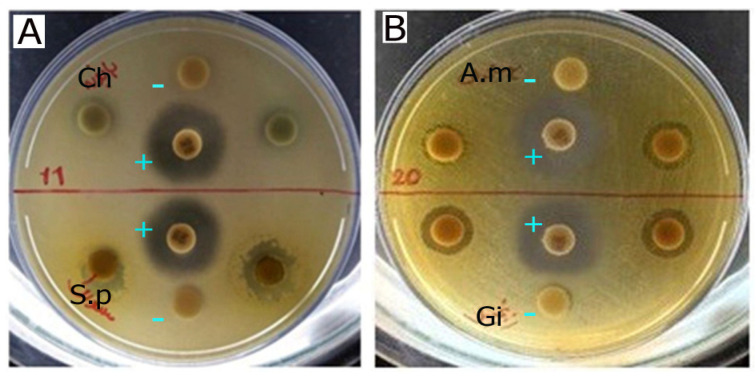
Each plate was divided into upper and lower parts, with each part containing a distinct extract. In the agar-plate (**A**), the upper section displays the inhibitory effect of *Matricaria chamomilla* extract (Ch) against *C. albicans* growth, while the lower section shows that of *Salvadora persica* extract (S.p). In the agar-plate (**B**), the upper section features *Achillea millefolium* extract (A.m) and the lower section features *Zingiber officinale* (ginger) extract (Gi). Nystatin (+) was used as the positive control, and DMSO (−) served as the negative control. Duplicate tests were conducted by placing two tablets on the right and left sides of each section.

**Figure 2:**
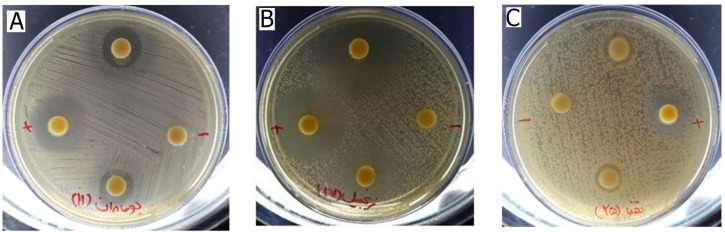
Inhibitory effects of *Achillea millefolium* essential oils (**A**), *Zingiber officinale* essential oils (**B**), and *Mentha spicata* essential oils (**C**) on the growth of *C*. *albicans*. Nystatin (+) was used as a positive control, while DMSO containing 0.5% (*v*/*v*) Tween 80 (−) served as the negative control. Duplicate tests were performed for each essential oil by placing two tablets (upper and lower sections) on each plate.

The antifungal activity observed in the MIC assays was further supported by the agar disc diffusion results. Figure 1 and Figure 2 show representative inhibition zones produced by the tested essential oils and extracts. Inhibition zone diameters are presented as ranges (mm) based on duplicate measurements (Table S1). Consistent with the MIC data, essential oils generally produced larger inhibition zones than the corresponding methanolic extracts. Among the tested samples, *Zingiber officinale* essential oil showed the strongest antifungal activity, as reflected by the largest inhibition zones.

### 2.5. Chemical Profile of Zingiber officinale Essential Oil

Given that *Zingiber officinale* essential oil exhibited the strongest antifungal activity among all tested samples, GC–MS analysis was performed to characterize its chemical composition and provide insight into the observed bioactivity. The hydrodistillation of fresh *Z*. *officinale* rhizomes yielded 0.62% (*v*/*w*, based on fresh weight) of pale-yellow essential oil. GC–MS analysis of the essential oil identified 12 major volatile constituents representing the predominant fraction of the oil composition. The essential oil was mainly composed of sesquiterpene hydrocarbons (SHs) and monoterpene hydrocarbons (MHs), indicating a terpene-rich profile characteristic of ginger oil. Zingiberene, a signature sesquiterpene of ginger, was identified as the most abundant compound, accounting for 21.49% of the total composition (Table 2). Other major constituents included sabinene (10.77%, MH), β-sesquiphellandrene (9.32%, SH), α-curcumene (8.16%, SH), and α-citral (geranial; 6.81%, oxygenated monoterpene). Additional compounds detected in notable proportions were octadienal (4.97%), α-farnesene (3.60%, SH), l-α-pinene (2.92%, MH), β-bisabolene (2.64%, SH), borneol (2.38%, oxygenated monoterpene), α-terpineol (1.33%, oxygenated monoterpene), and β-myrcene (1.26%, MH). Overall, the predominance of sesquiterpenes and monoterpenes suggests that these bioactive volatile constituents may contribute to the strong antifungal activity observed for ginger essential oil.

## 3. Discussion

Herbal medicines have gained attention for their broad biological activities—including antimicrobial, anti-inflammatory, and analgesic effects—with minimal side effects. In this study, the antifungal activity of extracts and essential oils from *S. persica*, *M. spicata*, *A. millefolium*, *M. chamomilla*, and *Z. officinale* was evaluated against *C. albicans* isolates obtained from denture stomatitis patients, using nystatin as a positive control. Overall, essential oils exhibited stronger antifungal effects than the corresponding methanolic extracts. Among the tested samples, *Z. officinale* essential oil showed the highest level of activity in both the broth microdilution and agar disc diffusion assays. A consistent relationship was observed between the two methods, with samples displaying lower MIC values generally producing larger inhibition zones. This agreement between quantitative and qualitative assessments supports the reliability of the observed antifungal activity.

The strong antifungal activity of *Z. officinale* essential oil observed in this study is in line with previous reports highlighting its broad antimicrobial potential. Ginger is widely recognized for its antioxidant properties [16] and has been reported to exhibit activity against a range of microorganisms, including *S. aureus*, *Enterococcus* spp., *Pseudomonas* spp., and *C. albicans* [17,18]. More recently, attention has focused on the antifungal potential of its essential oil [19,20]. Several mechanisms have been proposed to explain these effects. Bioactive constituents such as flavonoids, tannins, and alkaloids may interfere with fungal efflux pump systems and promote apoptotic processes [21,22]. In addition, ginger oleoresin has been reported to disrupt fungal membrane integrity through mechanisms such as electrolyte leakage and lipid peroxidation [23]. The findings of the present study are consistent with these observations, further supporting the potent antifungal activity of ginger-derived products. In contrast, *A. millefolium* extract showed relatively limited efficacy against *C. albicans* [24]. Moreover, the anti-*Candida* activity of ginger appears to be particularly pronounced, in some cases exceeding its general antimicrobial effects [25]. Notably, the activity of ginger essential oil was comparable to, and in some instances greater than, that of nystatin, including effects against fluconazole-resistant strains [25,26].

Nystatin exerts its antifungal effect by binding to membrane sterols and disrupting fungal cell integrity [27]. However, its broad pore-forming mechanism may also affect host cells, raising concerns regarding toxicity. Although *A. millefolium* essential oils have demonstrated some biological activity, the antifungal efficacy of its extracts appears inconsistent. Similarly, *S. persica* extracts showed variable antifungal effects depending on the extraction solvent used [28,29]. This variability may be attributed to differences in the presence of bioactive compounds such as sodium chloride, linoleic acids, and alkaloids, which have been associated with antimicrobial activity [30,31], as well as reported effects on biofilm formation and inflammation [32]. In comparison, *M. spicata* exhibited relatively weak antifungal activity compared with nystatin, and its efficacy remains insufficiently explored under standardized experimental conditions. Taken together, although nystatin remains an effective antifungal agent, its associated limitations—including potential toxicity and emerging resistance—highlight the need for alternative therapeutic strategies. In this context, the complex composition of essential oils may offer an advantage, as the combined or additive effects of multiple constituents can enhance overall antifungal activity.

The pronounced antifungal activity of *Z. officinale* essential oil observed in this study can be attributed to its complex, terpene-rich composition. GC–MS analysis showed that the oil is mainly composed of sesquiterpene and monoterpene hydrocarbons, with zingiberene (21.49%) identified as the predominant constituent. This profile is consistent with previously reported compositions of authentic ginger essential oils from different geographical origins [33,34], supporting the reliability of the present analysis. Sesquiterpenes such as zingiberene, β-sesquiphellandrene, α-curcumene, and α-farnesene contribute to the highly lipophilic nature of the oil. These non-polar compounds can readily interact with microbial lipid bilayers, leading to increased membrane permeability and structural disruption. As a result, leakage of intracellular components, impaired metabolic activity, and ultimately cell death may occur [35,36]. In addition to these hydrocarbons, oxygenated monoterpenes such as α-citral (geranial, 6.81%) and borneol (2.38%) are known for their strong anti-*Candida* activity [37,38]. Compared with hydrocarbon terpenes, oxygenated compounds are generally more reactive and may interfere with membrane-associated proteins, enzymatic pathways, and cellular redox balance. The coexistence of these two classes of compounds may further enhance antifungal efficacy through combined or synergistic effects, often described as an “entourage effect” [39]. Taken together, the interaction between membrane-active sesquiterpenes and biologically active oxygenated monoterpenes likely explains the strong anti-*C. albicans* activity of ginger essential oil observed in this study.

The essential oil yield of fresh *Z. officinale* rhizomes obtained in this study (0.62%, *v*/*w*, based on fresh weight) was higher than the 0.28–0.34% (*v*/*w*) range reported for fresh rhizomes extracted by hydrodistillation in previous studies [40], but lower than some values reported for dried rhizomes or optimized extraction conditions, which can reach approximately 1.0–3.0% [41]. These differences are likely attributable to variations in rhizome freshness, geographical origin, post-harvest handling, and extraction conditions. Collectively, these results indicate that the hydrodistillation method used in this study provides a moderate and reproducible yield within the expected range for fresh ginger rhizomes.

Although the clinical significance of *Candida* biofilms in denture stomatitis is well established, the present study evaluated antifungal activity only against planktonic *C. albicans* cells. It is important to note that biofilm-associated cells typically exhibit increased resistance to antifungal agents compared with planktonic counterparts. Therefore, the results of this study should be interpreted within the context of planktonic susceptibility, and further investigations using biofilm models are required to better assess the clinical applicability of the tested samples.

In addition, a limitation of this study is that detailed phytochemical profiling was conducted only for *Z. officinale* essential oil. The chemical composition of the other plant extracts and essential oils was not analyzed in this study and may vary depending on factors such as plant source and storage conditions. Therefore, comprehensive phytochemical characterization of all tested samples will be necessary in future studies to better establish the relationship between chemical composition and antifungal activity. In addition, the potential cytotoxic effects of *Z. officinale* essential oil on host cells were not assessed. Further investigations, including cytotoxicity studies using relevant mammalian cell models, will be essential to evaluate its safety and therapeutic applicability.

## 4. Materials and Methods

### 4.1. Preparation of Plant Extracts and Essential Oils

Fresh and dried parts of *Salvadora persica*, *Mentha spicata*, *Achillea millefolium*, *Matricaria chamomilla*, and *Zingiber officinale* were procured from a local market and authenticated by a pharmacognosy specialist at the Herbarium of the Faculty of Pharmacy, Tabriz University of Medical Sciences. To minimize variability in phytochemical composition, all plant materials were processed under standardized laboratory conditions immediately after procurement. Fresh samples were washed and air-dried when appropriate, and all materials were ground into fine powder and stored at 4 °C prior to extraction. Although detailed information regarding harvest time, cultivation conditions, and post-harvest handling was not available for the commercially sourced materials, all samples were handled consistently to ensure experimental reproducibility. Two extraction techniques were employed: maceration for methanolic extracts and hydrodistillation for essential oil isolation. For the preparation of methanolic extracts, 10 g of powdered plant material was extracted with 100 mL of methanol at room temperature (22–25 °C) for 24 h under occasional stirring. The mixture was subsequently filtered, and the solvent was removed under reduced pressure using a rotary evaporator (Heidolph, Germany). The dried extracts were stored at 4 °C until use. Essential oils were obtained by hydrodistillation using a Clevenger-type apparatus (Labbox, Germany). Briefly, 50 g of air-dried and ground plant material (*Achillea millefolium* and *Mentha spicata*) was subjected to hydrodistillation with 1000 mL of distilled water for 4 h. In particular, fresh ginger (*Zingiber officinale*) rhizomes were thoroughly washed, sliced into small pieces, and directly subjected to hydrodistillation for 4 h using a Clevenger-type apparatus. The collected essential oil was dried over anhydrous sodium sulfate, filtered, and stored in sealed amber glass vials at 4 °C until further chemical characterization and antimicrobial evaluation.

### 4.2. Clinical Fungal Isolation

A total of 25 patients with a clinical diagnosis of denture stomatitis (DS), as confirmed by the Faculty of Dentistry at Tabriz University of Medical Sciences, were randomly selected. Patients receiving medications, such as antibacterial or antifungal drugs, were excluded from the study. The research protocol was approved and registered by the Iran National Committee for Ethics in Biomedical Research (IR.TBZMED.REC.1397.221). Microbial samples were collected using sterile swabs (Sigma-Aldrich, St. Louis, MO, USA) and immediately cultured in tryptic soy broth (TSB) at both 25 °C and 37 °C for 24 to 48 h. The resulting cultures were subsequently transferred to Sabouraud dextrose agar plates (Sigma-Aldrich, St. Louis, MO, USA) containing 50 μg/mL of chloramphenicol to inhibit bacterial growth, and then incubated at 37 °C for 24 h. *Candida* isolates were initially cultured on Sabouraud dextrose agar and identified based on colony morphology. Species-level identification of *Candida albicans* was further confirmed using standard phenotypic methods, including germ tube formation and morphological characteristics.

### 4.3. Preparation of Antifungal Test and Determination of Minimum Inhibitory Concentrations (MICs)

The minimum inhibitory concentrations (MICs) of the plant extracts and essential oils against *Candida albicans* were determined using the broth microdilution method according to CLSI guidelines (M27-A3) [42]. Briefly, stock solutions of the dried methanolic extracts were prepared by dissolving the extracts in dimethyl sulfoxide (DMSO) at a concentration of 100 mg/mL. The solutions were vortexed and, when necessary, sonicated to ensure complete dissolution. The stock solutions were subsequently diluted with the culture medium to obtain the desired concentrations, and the final concentration of DMSO in each well did not exceed 1% (*v*/*v*). Essential oils were initially dissolved in DMSO and subsequently diluted in broth medium containing 0.5% (*v*/*v*) Tween 80 as a non-ionic emulsifying agent to ensure uniform dispersion. The mixtures were vortexed thoroughly prior to use. The final concentration of DMSO in each well did not exceed 1% (*v*/*v*). Serial two-fold dilutions of each test substance were prepared in 96-well microtiter plates over a concentration range of 6.25–100 mg/mL in appropriate broth medium. A standardized fungal inoculum (1 × 10^6^ CFU/mL) was prepared from freshly cultured colonies and 100 μL was added to each well to achieve the final test concentration. Plates were incubated at 37 °C for 24 h. The MIC was defined as the lowest concentration at which no visible fungal growth was observed. Nystatin was used as the positive control, while the corresponding solvent control containing DMSO (and Tween 80 for essential oil preparations) without test sample served as the negative control. All experiments were performed in triplicate, and the results were consistent across independent repetitions.

### 4.4. Agar Disc Diffusion Assay

For the agar disc diffusion assay, a standardized *C. albicans* suspension (1 × 10^6^ CFU/mL) was uniformly spread onto tryptic soy agar plates. Sterile paper discs (6 mm in diameter) were impregnated with 10 μL of each sample solution (100 mg/mL), corresponding to 1 mg/disc, and placed on the inoculated agar surface. Nystatin-loaded discs were used as the positive control at the same loading volume (10 μL/disc), while discs loaded with the corresponding solvent system [DMSO or DMSO containing 0.5% (*v*/*v*) Tween 80, as appropriate] served as the negative control. After incubation at 37 °C for 24 h, inhibition zone diameters were measured in millimeters, including the diameter of the disc. All experiments were performed in duplicate.

### 4.5. Gas Chromatography–Mass Spectrometry (GC–MS) Analysis of Ginger Essential Oil

The chemical composition of *Zingiber officinale* essential oil (GEO) was analyzed using an Agilent 6890N gas chromatograph (Agilent, Santa Clara, CA, USA) coupled with an Agilent 5973N mass selective detector (Agilent, Santa Clara, CA, USA) [43,44,45]. The essential oil sample was diluted in *n*-hexane prior to analysis, and 1 μL of the diluted sample was injected in split mode (split ratio 1:50). Separation was performed on an Rxi-5MS capillary column (30 m × 0.25 mm i.d., 0.25 μm film thickness, Restek, Bellefonte, PA, USA). The oven temperature was initially set at 70 °C and held for 3 min, then increased to 240 °C at a rate of 10 °C/min and maintained at this temperature for 5 min. Helium was used as the carrier gas at a constant flow rate of 1.0 mL/min. The injector temperature was set at 150 °C. Mass spectra were obtained by electron ionization (EI) at 70 eV over a mass range of *m*/*z* 40–550. Identification of the constituents was carried out by comparison with the NIST library database and by calculation of retention indices (RIs). The relative composition (%) of the essential oil components was determined based on peak area normalization without the use of an internal standard.

## 5. Conclusions

In this study, 25 clinical isolates of *C. albicans* obtained from denture stomatitis patients were evaluated for susceptibility to plant-derived methanolic extracts and essential oils. Overall, essential oils exhibited significantly stronger antifungal activity than their corresponding extracts. Among the tested preparations, *Z. officinale* essential oil demonstrated the most potent anti-*C. albicans* activity, inhibiting 15 out of 25 isolates and showing MIC values ranging from 6.25 to 50 mg/mL, which were comparable to or lower than those of nystatin in several cases. GC–MS analysis revealed that the antifungal activity of ginger essential oil is likely associated with its terpene-rich composition, with zingiberene (21.49%) being the predominant component, followed by sabinene (10.77%), β-sesquiphellandrene (9.32%), and α-curcumene (8.16%). Taken together, these findings suggest that ginger essential oil represents a promising natural antifungal candidate for the management of denture-associated candidiasis. However, further studies including biofilm models, toxicity evaluation, and in vivo validation are warranted before clinical application.

## Figures and Tables

**Table 1:** Antifungal activity and minimum inhibition concentration (mg/mL) of extracts and essential oils against *C. albicans* ^a^.

Case No.	*Z. officinale* Essential Oil	*Z. officinale* Extract	*A. millefolium* Essential Oil	*A. millefolium* Extract	*M. chamomilla* Extract	*M. spicata* Essential Oil	*S. persica* Extract	Nystatin
1	50	100	–	–	50	–	–	25
2	–	–	–	–	–	–	–	50
3	–	–	–	–	–	-–	–	25
4	–	–	–	–	50	50	–	18.75
5	–	–	–	–	–	–	–	25
6	–	–	–	–	–	–	–	25
7	–	–	–	–	–	–	–	–
8	50	100	–	–	–	50	–	50
9	–	–	–	–	–	37.5	–	25
10	25	100	–	–	100	–	–	25
11	37.5	100	50	100	100	50	25	50
12	50	37.5	–	–	–	–	–	37.5
13	25	50	–	–	–	–	–	25
14	37.5	75	37.5	75	–	–	75	25
15	12.5	100	–	–	–	–	–	25
16	37.5	37.5	–	–	–	–	–	50
17	6.25	37.5	–	–	–	–	–	25
18	25	37.5	–	–	–	–	–	25
19	18.75	100	–	–	–	–	–	25
20	12.5	25	–	75	–	50	–	25
21	9.375	18.75	–	–	–	–	–	25
22	50	100	18.75	100	–	–	–	25
23	–	–	–	–	–	–	–	50
24	–	–	–	–	–	–	–	25
25	–	–	–	–	–	50	–	37.5

*^a^* “–“ indicates no detectable antifungal activity at the tested concentrations.

**Table 2:** Chemical composition of *Zingiber officinale* essential oil identified by GC–MS analysis ^a^.

Components	Area%	CAS Number	RT
β-Myrcene	1.26	123-35-3	5.948
α-Terpineol	1.334	98-55-5	9.699
Borneol	2.384	507-70-0	9.211
β-Bisabolene	2.64	495-61-4	16.479
L-α-pinene	2.921	7785-26-4	5.016
α-Farnesene	3.603	502-61-4	16.39
Octadienal	4.97	106-26-3	10.864
α-Citral	6.808	141-27-5	11.519
α-curcumene	8.161	644-30-4	15.857
β-sesquiphellandrene	9.321	307-83-9	16.845
Sabinene	10.77	3387-41-5	6.758
Zingiberene	21.493	95-60-3	16.246

**^a^** The relative composition (%) of each compound was calculated based on peak area normalization; RT: retention time (min).

## Data Availability

The original contributions presented in this study are included in the article. Further inquiries can be directed to the corresponding author.

## Supplementary Materials

The following supporting information can be downloaded at https://www.mdpi.com/article/10.3390/plants15091392/s1. Table S1: Inhibition zone diameters (mm) of plant extracts and essential oils against *C. albicans*.
