# Supplementary material for: Comparative Antifungal Activity of Medicinal Plant Extracts and Essential Oils Against Clinical Isolates of Candida albicans from Denture Stomatitis Patients
Source: Plants (Basel). 2026 May 1;15(9):1392. doi: 10.3390/plants15091392 (PMC13164761; doi:10.3390/plants15091392)
Supplement: Supplementary file 1 [file plants-15-01392-s001.zip › plants-4187477-supplementary.pdf]

Comparative Antifungal Activity of Medicinal Plant Extracts and Essential Oils against Clinical Isolates of *Candida albicans* from Denture Stomatitis Patients

Nazanin Fathi [1,†], Joo-Hyun Hong [2,†], Farzaneh Lotfipour [3], Samin Ghaffari [4], Reza Abbasi [5], Parina Asgharian [6], Rana Attaran [5], Hamed Hamishehkar [7,8], Maryam Kouhsoltani [5,*], Ki Hyun Kim [2,*]

[1] Research Center for Immunodeficiencies, Pediatrics Center of Excellence, Children's Medical Center, Tehran University of Medical Sciences, Tehran, Iran; nazaninfathi26@gmail.com
[2] School of Pharmacy, Sungkyunkwan University, Suwon 16419, Republic of Korea; ehong@skku.edu
[3] Food and Drug Safety Research Center, and Department of Pharmaceutical and Food Control, Faculty of Pharmacy, Tabriz University of Medical Sciences, Tabriz, Iran; farzaneh.lotfipour@gmail.com
[4] Department of orthodontics, Faculty of Dentistry, Shahid Beheshti University of Medical Sciences, Tehran, Iran; saminghaffari@gmail.com
[5] Department of Oral and maxillofacial Pathology, Faculty of Dentistry, Tabriz University of Medical Sciences, Tabriz, Iran; reza.abbasy20@gmail.comrana.ataran@gmail.com (Re. A.); rana.ataran@gmail.com (Ra. A.)
[6] Department of Pharmacognosy, Faculty of Pharmacy, Tabriz University of Medical Sciences, Tabriz, Iran; parina.asgharian@gmail.com
[7] Drug Applied research Center, Tabriz University of Medical Sciences, Tabriz, Iran; hamishehkar.hamed@gmail.com
[8] New Material and Green Chemistry Research Center, Khazar University, 41 Mehseti Street, Baku, AZ1096, Azerbaijan
* Correspondence: mkoohsoltani@yahoo.com, koohsoltanim@tbzmed.ac.ir (M.K.); khkim83@skku.edu (K.H.K.)
† These authors contributed equally to this work.

Table S1. Inhibition zone diameters (mm) of plant extracts and essential oils against *C. albicans*.[a]

Sample	Zone of inhibition (mm)
Matricaria chamomilla extract	6.8 - 7.0
Salvadora persica extract	8.2 - 9.1
Achillea millefolium extract	6.6 - 8.1
Zingiber officinale extract	8.7 - 9.0
Achillea millefolium essential oils	9.5 - 11.2
Zingiber officinale essential oils	18.3 - 20.4
Mentha spicata essential oils	8.2 - 8.8
Nystatin	17.2 - 21.5

[a]Values are expressed as ranges based on duplicate measurements (n = 2); Nystatin was used as the positive control.
